# Radiotherapy for Melanoma: More than DNA Damage

**DOI:** 10.1155/2019/9435389

**Published:** 2019-04-03

**Authors:** Susanne J. Rogers, Emsad Puric, Brigitte Eberle, Niloy R. Datta, Stephan B. Bodis

**Affiliations:** ^1^Centre for Radiation Oncology KSA-KSB, Kantonsspital Aarau, Aarau 5001, Switzerland; ^2^Department of Radiation Oncology, University Hospital Zurich, Zurich 8091, Switzerland

## Abstract

Despite its reputation as a radioresistant tumour, there is evidence to support a role for radiotherapy in patients with melanoma and we summarise current clinical practice. Melanoma is a highly immunogenic tumour and in this era of immunotherapy, there is renewed interest in the potential of irradiation, not only as an adjuvant and palliative treatment, but also as an immune stimulant. It has long been known that radiation causes not only DNA strand breaks, apoptosis, and necrosis, but also immunogenic modulation and cell death through the induction of dendritic cells, cell adhesion molecules, death receptors, and tumour-associated antigens, effectively transforming the tumour into an individualised vaccine. This immune response can be enhanced by the application of clinical hyperthermia as evidenced by randomised trial data in patients with melanoma. The large fraction sizes used in cranial radiosurgery and stereotactic body radiotherapy are more immunogenic than conventional fractionation, which provides additional radiobiological justification for these techniques in this disease entity. Given the immune priming effect of radiotherapy, there is a strong but complex biological rationale and an increasing body of evidence for synergy in combination with immune checkpoint inhibitors, which are now first-line therapy in patients with recurrent or metastatic melanoma. There is great potential to increase local control and abscopal effects by combining radiotherapy with both immunotherapy and hyperthermia, and a combination of all three modalities is suggested as the next important trial in this refractory disease.

## 1. Introduction

Malignant melanoma is reputed to be a radioresistant tumour but there are historical reports of successful empirical irradiation of black naevi with little skin toxicity shortly after the discovery of x-rays and series from the 1960's reporting 5-year survival rates equivalent to surgery [1]. As wide local excision became established as the primary therapy for melanoma and radiobiological experiments* in vitro* reported low radiation sensitivity, radiotherapy played a minor role in the management of patients with melanoma until further laboratory data showed induction of DNA damage after irradiation and hence radiation sensitivity in at least some cell lines [2].

### 1.1. Hypofractionation

Radiotherapy is conventionally prescribed at 2 Gy per treatment (fraction). When larger daily doses are administered, this is termed hypofractionation. Early clinical series reported up to double the complete response rate when more than 4 Gy per fraction was delivered [3] and formed the basis for the early randomized controlled trials exploring fraction size. 8 x 5 Gy twice a week in the control arm was compared against 3 x 9 Gy twice a week [4]. An impressive 97% overall response rate was achieved with no difference in either efficacy or toxicity between the two arms. Similarly, the RTOG 83-05 study [5] closed early as no difference in response rates between 20 x 2.5 Gy daily and 8 x 4 Gy was detectable. Radiobiological data pertaining to the likely low *α*/*β* ratio (i.e., increased sensitivity to large fraction size) [6], the wealth of retrospective data, and the patient convenience of fewer fractions has led to a typical hypofractionated dose prescription of between 5 and 9 Gy per fraction. Technical advances in radiotherapy delivery (3D conformal, intensity-modulated, radiosurgery, proton therapy, and brachytherapy) enable a higher radiation dose per fraction to be delivered routinely without additional normal tissue damage.

### 1.2. Radiation as an Alternative or Addition to Surgery

A particular advantage of radiation over surgery is in the primary therapy of choroidal melanoma, where irradiation with protons can avoid enucleation and achieve local control rates exceeding 95% and a 5-year survival similar to surgery [7, 8]. Radiotherapy is also highly effective as primary therapy for in situ melanoma (lentigo maligna) with only 5% recurrence rates and 1.4% progression to malignant melanoma [9]. However, according to the National Comprehensive Cancer Network (NCCN) management guidelines, primary irradiation of a cutaneous melanoma is only recommended in medically inoperable patients or if wide local resection would be associated with unacceptable morbidity.

Postoperative irradiation of the primary site is not standard practice as increasing awareness of melanoma has led to the diagnosis of earlier stage tumours and wide local excision can achieve 95% local control rates. Adjuvant irradiation should be considered in cases of invasive melanoma with positive histological margins despite optimal surgery or desmoplastic histology with margins <1cm where reresection is not feasible and/or with extensive neurotropism [10]. A single arm phase II trial (NCCTG N0275) reported 90% local control at 2 years following 5 x 6 Gy to completely resected desmoplastic melanomas, hypothesising a role for radiotherapy in all patients with this subtype, regardless of margin [11]. Furthermore, randomised trial data support postoperative radiotherapy for melanoma patients with a high risk of lymph node relapse. The landmark TROG study (ANZMTG 01.02/TROG 02.01) reported a 36% nodal relapse rate after 6 years of observation following lymph node dissection, which was reduced to 21% by postoperative nodal radiotherapy (PORT), an odds ratio of 0.52 without any impact on overall survival [12]. PORT to the cervical and axillary nodes was not associated with toxicity; however, PORT to the inguinal lymph nodes doubled the observed incidence of leg lymphedema from 7% after surgery alone to 15%. The nodal risk factors associated with a clinical benefit from PORT are shown in Table 1.

Mucosal melanoma carries a worse prognosis than the cutaneous form and the benefit of adjuvant radiotherapy has been controversial. A meta-analysis of over 1593 patients with head and neck mucosal melanoma from 12 studies reported a highly significant hazard ratio of 0.51 (95% CI 0.35-0.76, p=0.155) in favour of PORT, again with no survival advantage [13]. The widespread availability of intensity modulated radiotherapy (IMRT) enables a considerable reduction in radiation-associated normal tissue toxicity seen previously and thus facilitates nodal and mucosal PORT when indicated.

### 1.3. Palliative Radiotherapy

Radiotherapy in patients with melanoma is most frequently delivered in the palliative setting, particularly for nodal, satellite, and in-transit metastases that are unresectable or have progressed despite systemic therapy, and for the management of brain metastases. Historically, whole brain radiotherapy (WBRT) was standard practice, but the QUARTZ trial has shown that patients with poor performance status and short life expectancy do not benefit more from WBRT than from steroids alone [14]. Due to the lack of survival advantage and potential neurocognitive toxicity, WBRT is today viewed as a last resort and several WBRT trials have closed early due to poor accrual [15, 16]. Sparing the hippocampus is both technically feasible and clinically acceptable as less than 5% of melanoma brain metastases arise in the hippocampi. Phase II data suggest protecting the hippocampi from irradiation during WBRT preserves short-term memory and may bring WBRT back into favour for patients with numerous brain metastases if the neurocognitive toxicity can be thus offset [17, 18]. A patient with a B-RAF v600 mutation and small volume asymptomatic brain metastases may be treated with an oral tyrosine kinase inhibitor in the first instance; however, frequent imaging should be performed to offer timely salvage with irradiation.

### 1.4. Stereotactic Cranial and Extracranial Radiosurgery

The potential neurocognitive deficits (short-term memory loss, delayed recall), fatigue, and alopecia along with the lack of survival benefit associated with WBRT are the compelling rationale for radiosurgery for brain metastases. Stereotactic radiosurgery (SRS) is the terminology for the very precise delivery of high dose irradiation to small volumes in one to six sessions (fractions) with a steep dose fall-off outside the irradiated volume. SRS is now well established as a nonsurgical treatment alternative for brain metastases when histological confirmation is not required, there is no mass effect, or the lesion is not surgically accessible. A series with over 300 melanoma brain metastases reported one year local control rates of 91% following radiosurgery, not inferior to other histologies [19]. Hypofractionated stereotactic radiotherapy (SRT) in 3-6 fractions is reported to achieve very similar local control rates to single fraction ablative doses for melanoma brain metastases under 3cm in diameter [20] suggesting a relative radiosensitivity of melanoma or, at least, that the nonablative dose is offset by the tumour cell reoxygenation and cell cycle redistribution during the course of treatment. By extension, SRS/SRT are increasingly employed following resection of a brain metastasis as single or multiple fractions instead of whole brain radiotherapy [21].

There is a growing momentum behind stereotactic body/ablative radiotherapy (SBRT or SABR) for the control of oligometastases (up to five metastases at less than three metastatic sites). Local ablative irradiation is an attractive noninvasive and relatively nontoxic option for the management of oligorecurrence (small volume metastatic disease with a controlled primary), or oligoprogression in the case of a mixed response to defer the next line of systemic therapy. Typical SBRT fraction sizes range from 9 to 20 Gy and retrospective series including melanoma patients have shown the utility of this therapeutic modality [22]. A phase I/II trial of patients with liver metastases of various histologies treated with 60 Gy in 3 fractions showed an impressive 2-year local control rate of 100% for lesions of ≤ 3 cm [23], but an impact on survival has yet to be demonstrated.

### 1.5. Radiation and Hyperthermia

**Figure 1:**
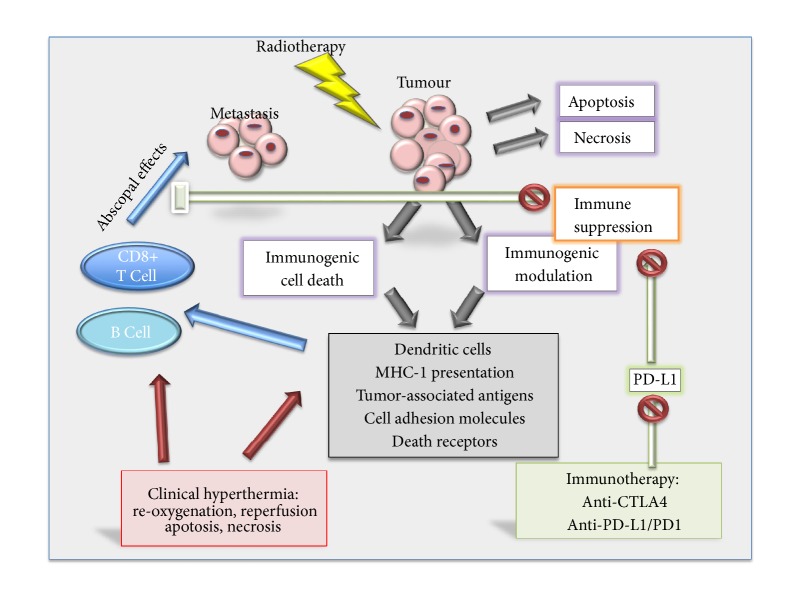
Schematic of the potential costimulation of the immune system by radiotherapy, immune therapy, and hyperthermia.

Radiation induces not only single- and double-strand DNA strand breaks resulting in apoptosis and necrosis, but also immunogenic modulation. Immunogenic cell death leads to the release of danger-associated molecular patterns (DAMPs) including calreticulin and ATP. Such DAMPs both recruit and activate dendritic cells to take up and to cross-present tumour antigens to naïve T cells thus initiating antitumour immune responses [24]. Furthermore, cell adhesion molecules, death receptors, and tumour-associated antigens are also released (Figure 1); thus, the tumour can effectively be transformed into an individualised vaccine. The cellular response to radiation is complex; and particularly under hypoxic conditions, immunosuppressive mediators including HIF-1a, TGFb, and extracellular adenosine may also be released, increasing immune tolerance. The preexisting conditions in the tumour microenvironment and preponderance of tumour-associated macrophages and regulatory T-cells determine the extent of dendritic cell and effector T cell activation by radiation [24].

**Figure 2:**
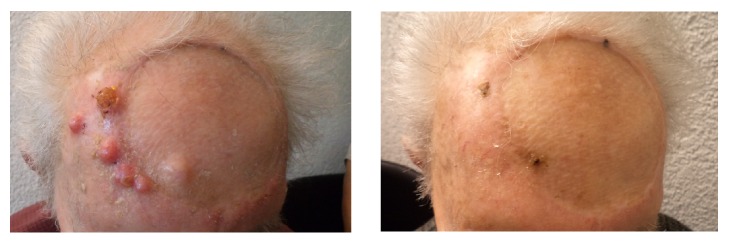
Complete clinical response of cutaneous malignant melanoma metastases three months following irradiation with 3 x 9 Gy with 80kV combined with weekly superficial hyperthermia.

**Figure 3:**
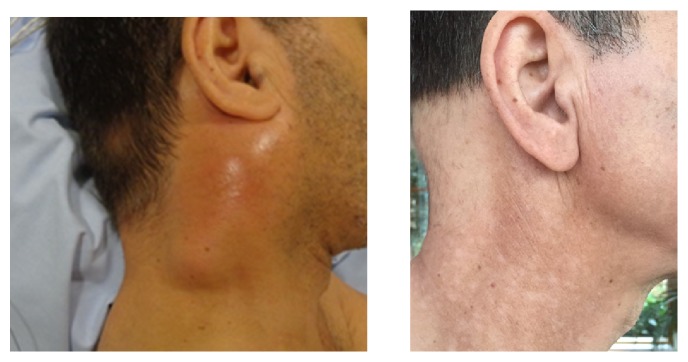
Complete clinical response of a cutaneous melanoma metastatic cervical lymph node conglomerate that developed during systemic therapy with nivolumab and ipilimumab, two months following irradiation with 12 x 3 Gy with 6MV over 3 weeks, combined with twice-weekly superficial hyperthermia.

Radiotherapy can achieve good palliation of metastases; however, the overall response rate can be significantly enhanced by combination with hyperthermia. The superior local control rates achieved at two years with combined radiation and hyperthermia (46%) as compared with radiation alone (28%) in patients with recurrent or metastatic disease in the randomised trial ESHO 3-85 are proof of concept for melanoma [25]. Heating a tumour to 43°C for 60 minutes once or twice a week during the course of radiation can achieve greater radiation responses due to enhanced perfusion and oxygenation, inhibition of DNA repair mechanisms, cell death, and broad immune modulation. This immune response includes increased expression of immunogenic surface receptors such as MHC-1 and secretion of heat shock proteins [26], which activate the natural killer and antigen presenting cells, thus increasing CD8+-mediated immune responses. The latter enable unirradiated tumour sites to respond clinically due to the migration of irradiation-induced immunogenic factors, the so-called abscopal effect. It has been demonstrated in a resected sarcoma treated with preoperative hyperthermia and radiotherapy that hyperthermia indeed recruited CD68 + macrophages to the tumour [27]. In our institution, we routinely treat metastatic melanoma with hypofractionated radiotherapy, e.g., 3 x 9 Gy twice weekly, and superficial hyperthermia at 43°C with better objective (tumour volume) and subjective (pain) clinical outcomes than expected from radiotherapy alone (Figures 2 and 3). It is important to achieve 43°C as hyperthermia at 41.5°C appeared to strengthen rather than weaken radiation-induced S and G2 DNA repair checkpoints [28].* In vitro* evidence suggests a greater immunogenic potential of melanoma cells following irradiation combined with hyperthermia, culminating in increased apoptotic and necrotic melanoma cells as compared with irradiation alone [29]. Several meta-analyses have shown the superiority of hyperthermia as a radiosensitiser in other solid tumour types, frequently more efficient than classical chemotherapy and with considerably less toxicity [30–32].

### 1.6. Immunotherapy

Since 2011, the systemic treatment options for melanoma have expanded considerably from chemotherapy with limited efficacy, to tyrosine kinase inhibitors (vemurafenib, dabrafenib) for patients with a B-RAF v600 mutation, augmented by a MEK inhibitor (trametinib) [33], and T-cell checkpoint inhibitors such as ipilimumab, nivolumab, and pembrolizumab. Molecular characterisation for BRAF, NRAS, cKIT, and p53 mutations and PD-L1 biomarker expression levels are now part of routine diagnostic melanoma pathology in our institution. Despite the immunogenicity of melanoma, abscopal effects are thought to be rare due to the relative immune suppression in a tumour microenvironment. Immune tolerance results from the binding of programmed death-ligand 1 (PD-L1) to the programmed death receptor -1 (PD-1), expressed on T-cells and pro-B cells [34]. This ligand-receptor interaction transmits an inhibitory signal that reduces antigen-specific T-cell proliferation and inhibits regulatory T-cell apoptosis [35], the so-called T-cell checkpoint inhibition. PD-L1 expression can be detected on tumour cells, either constitutively as part of carcinogenesis or induced by a T-cell infiltrate [36], and is used to select patients with an increased probability of responding to immunotherapy.

Ipilimumab was the first FDA-approved CTLA-4 inhibitor. Its negative regulation of T-cell activation results in an enhanced cytotoxic antitumour T-cell response. Following complete resection of cutaneous melanoma, a randomised phase III trial showed a statistically significant increase in recurrence free survival: 46.5% with adjuvant ipilimumab over 34.8% with placebo, but drug-related adverse events led to discontinuation of treatment in 52% of patients and death in 1% of patients [37]. The PD-1 inhibitors nivolumab and pembrolizumab display more favourable efficacy and toxicity profiles and have superseded single agent ipilimumab [38, 39]. Superior efficacy of combined nivolumab and ipilimumab over the latter alone has been demonstrated in mucosal [40] and unresectable cutaneous melanoma [41] but the combination may be offered in the metastatic setting. A multidisciplinary tumour board today is more likely to recommend adjuvant systemic therapy with a checkpoint inhibitor than irradiation following resection of melanoma stages IIIB-IV [42].

A different approach to enhancing the immune detection and destruction of cancer cells is through vaccination [43]. Several types of vaccine are being researched including antigen, whole cell, dendritic cell, DNA, and anti-idiotype vaccines. Dendritic cell vaccines, for example, aim to overcome the immunosuppression in the tumour microenvironment and mount a specific anticancer response [44], and an increase in median overall survival in patients who showed a delayed hypersensitivity response (22.8 months) as compared with those who did not (4.8 months) [45] is proof of principle of this therapeutic strategy in stage IV melanoma.

### 1.7. Radiotherapy Research: Combined Modality Therapy

Currently, the main radiation oncology research focus in patients with melanoma is the development of safe and efficacious combined modality treatments. There have been some very serious toxicities including death following radiosensitisation by concomitant B-RAF TKi with SBRT [46, 47] and the NCCN and ECOG guidelines are to withhold a BRAFi and/or MEKi 3 days before and after fractionated radiotherapy and 1 day before and after SBRT/SRS [48]. There is therefore greater scope to combine radiation with immune checkpoint inhibitors, particularly as radiation and checkpoint inhibitors activate the immune system through nonredundant mechanisms [49]. Each modality has limited activity alone; for example, less than 50% of melanoma tumours have the CD8+ T cells that are requisite for response to PD-1 inhibition [50]. Given the immune priming effect of RT, there are both a strong but complex biological rationale and an increasing body of evidence for synergy in combination, recently reviewed in depth [51–53]. There are several case reports of presumed abscopal responses [54–56]; however, delayed responses to immunotherapy after initial tumour flare are difficult to differentiate [57]. Several immunoradiotherapy trials have chosen ‘out of field' responses as a novel primary endpoint [50]. For example, the ‘PERM' phase II trial randomises patients with melanoma between a control arm of pembrolizumab against pembrolizumab with 3 x 8 Gy radiotherapy (excluding brain and abdomen) to assess not only any increase in local control rates but also abscopal responses [58].

The published studies of ipilimumab combined with irradiation have been recently summarised [59]; however, the programmed death-1 (PD-1) inhibitory antibodies such as pembrolizumab and nivolumab now appear to be the main research focus and prospective data are needed. According to the clincialtrials.gov database, around 50 trials are currently investigating combinations of radiotherapy with immunotherapy in melanoma. Recently, a phase I trial reported successful delivery of SBRT in patients who also received immunotherapy with ipilimumab [60]. Two prospective studies of PD-1 pathway inhibition combined with body or cranial irradiation have also been published [61, 62]. Importantly, there did not seem to be any increase in the incidence or severity of toxicity over and above that seen with each modality alone; however, the need for novel combinations to be evaluated for safety before widespread adoption has been recently highlighted in a current trial of hypofractionated radiotherapy for bladder cancer (6 x 6 Gy) with pembrolizumab where dose-limiting toxicity was reported and the fraction size will be decreased [63].

Immune therapies have very long half-lives and it is inevitable that the immune response will have been initiated and still be active at the time of radiosurgery if immunotherapy has already begun. A randomised trial without immunotherapy in the control arm is now unlikely in recurrent or metastatic melanoma; thus, patient data registries such as the ongoing German radiation oncology association (DEGRO) ‘TOaSTT' registry, established to collect toxicity data from patients treated with targeted therapies within 30 days before or after cranial or extracranial radiosurgery, will be valuable to ascertain the toxicities seen in practice.

As well as selecting the best systemic agent(s) and optimising timing of delivery (concurrent or sequential), the optimal radiation fractionation must also be established. It has been shown that BED 100 Gy_10_ is more immunogenic than conventional fractionation and that fraction sizes above 12 Gy are necessary to induce immune effects [64–66].* In vivo* studies suggest a threshold dose of 12-18 Gy in a single fraction, after which larger fraction sizes are less immunogenic than smaller doses per fraction [67]. Ipilimumab combined with 4 x 12.5 Gy is reported to be safe in patients with toxicities not exceeding those expected from either treatment modality alone [68]. Interestingly, the site of irradiation also plays a role as SBRT of liver metastases appears more immunogenic than that of lung metastases as determined by the increased production of T-cells expressing proimmune antigens [68].

### 1.8. Brain Metastases

As described above, control rates of 60-90% at one year have been reported with SRS for melanoma brain metastases. There is some potential to increase local control and a definite need to reduce distant brain relapse, hence the numerous current studies of combined modality therapies in melanoma patients with brain metastases. Combinations with B-RAF TKIs and the checkpoint inhibitors are predominantly being explored. In a retrospective study, median survival was greater in patients who received a B-RAF TKI after, rather than during or before, SRS for brain metastases but the incidence of intracranial haemorrhage was higher (10.4%) in the B-RAF TKI group as compared with 3% in the those who did not receive the targeted therapy [69]. In a retrospective series, the combination of ipilimumab and radiosurgery for brain metastases was reported to show higher rates of response over SRS alone; however, the combination was also associated with increased haemorrhage and treatment-related imaging changes (TRICs) [70]. Provocatively, the analysis of a subgroup of melanoma patients with brain metastases only did describe a marked increase in median survival (56.4 mths vs 7.7 mths) and 4-year OS (51.5% vs 16.9%), with checkpoint blockade immunotherapy, however, suggesting significant intracranial efficacy [71]. A 46% response rate, in the context of a 54% grade 3-4 treatment-related adverse event rate, has been reported with combined nivolumab and ipilimumab for asymptomatic melanoma brain metastases [72].

Also of interest in the therapy of melanoma brain metastases is temozolomide, an established radiosensitiser in primary brain tumours through its inhibition of MGMT, a DNA repair protein. In addition, temozolomide is metabolised to dacarbazine, one of the chemotherapeutics used in melanoma, and demonstrates selective depletion of regulatory T cells in vitro, thus offering, in theory, a triple-pronged attack. Results to date are modest however [73]. Many systemic agents are being explored but perhaps the most exciting recent report pertains to talimogene laherparepvec (T-vec), the first FDA approved oncolytic viral therapy. In combination with pembrolizumab and WBRT, T-vec is reported to have achieved complete response of numerous brain metastases in patients with melanoma previously resistant to ipilimumab and nivolumab [74].

The radioresistant reputation of melanoma and nihilistic perception of the prognosis of patients with metastatic disease prior to the availability of tyrosine kinase inhibitors and immune therapies are probable reasons behind the lack of recent clinical trials of radiation with hyperthermia in this disease entity. Ongoing preclinical research aims to deepen our mechanistic understanding [29]. Recent significant meta-analyses consistently documenting 20-30% improvement in clinical outcome as compared with radiation or chemoradiation in breast, head and neck, and cervix cancers [30, 31, 75] and the strong radiobiological rationale for combining hyperthermia with protons [76] or with nanotechnology [77, 78] have rekindled interest in clinical trials in hyperthermia for refractory diseases [79, 80]. Important research questions to be examined in future randomised clinical trials in recurrent or metastatic melanoma include whether the addition of superficial hyperthermia achieves higher local control rates and/or rates of abscopal response in combination with radiation and immunotherapy. Possible trial designs could be based on 3 x 9 Gy, the recommended dose from ESHO 3-85 [25], in combination with pembrolizumab (as in the PERM trial) [58], randomised to hyperthermia and radioimmunotherapy or to radioimmunotherapy alone. An alternative would be radiation combined with nivolumab and ipilimumab randomised to additional hyperthermia. The nonredundant immune system activation and apparently noncumulative toxicities seen with irradiation, hyperthermia, and immunotherapy strongly suggest that a combination of all three modalities might benefit melanoma patients with extracranial melanoma recurrences or metastases.

## 2. Conclusions

Whilst historically the role of radiation in the management of melanoma has been questioned due to perceived radioresistance, data and clinical experience support radiotherapy for cranial and extracranial palliative indications, the adjuvant irradiation of primary cutaneous and mucosal tumours at high risk of local recurrence, and the adjuvant therapy of patients at high risk of nodal recurrence. Radiotherapy can be more effective when delivered as radiosurgery or when combined with hyperthermia and the combination of radiotherapy with immunotherapy may increase the real but elusive abscopal effects. As radiation and hyperthermia are well tolerated, and radiation and immunotherapy generally do not show additive toxicity, a future trial evaluating radiation combined with both hyperthermia and checkpoint inhibition could be important for patients with relapsed melanoma.

## Figures and Tables

**Table 1:** 

Nodal risk factors
Extracapsular spread
≥ 1 parotid node
≥ 2 cervical or axillary nodes
≥ 3 inguinofemoral nodes
≥ 3 cm cervical or axillary node
≥ 4 cm inguinofemoral node
